# Enhancing or Hindering? The Moderating Role of Self-Regulated Learning in the Relationship Between GenAI Usage and Higher-Order Thinking Skills Among Chinese University Students

**DOI:** 10.3390/jintelligence14090208

**Published:** 2026-09-01

**Authors:** Shuangshuang Li, Jin Tian, Mengting Chen, Ziyue Wang

**Affiliations:** Faculty of Education, Henan Normal University, Xinxiang 453000, China

**Keywords:** GenAI, self-regulated learning, higher-order thinking

## Abstract

The rapid integration of generative artificial intelligence (GenAI) into educational settings has raised growing concerns regarding its potential influence on students’ higher-order thinking skills. However, existing findings remain inconclusive, and the role of individual factors in shaping these effects has received limited attention. Drawing on self-regulated learning theory, this study examined the relationships between GenAI usage and three higher-order thinking skills, critical thinking, creative thinking, and computational thinking, and investigated the moderating role of self-regulated learning. The results indicated that the associations between GenAI use and higher-order thinking skills varied across levels of self-regulated learning. Specifically, among students with lower levels of self-regulated learning, GenAI use was negatively associated with critical thinking, creative thinking, and computational thinking. In contrast, among students with higher levels of self-regulated learning, GenAI use was positively associated with critical thinking and computational thinking, while its association with creative thinking was not significant. Furthermore, self-regulated learning was positively associated with all three higher-order thinking skills and significantly moderated the relationships between GenAI use and critical thinking, creative thinking, and computational thinking. These findings highlight the complex cognitive consequences of GenAI usage and underscore the importance of self-regulated learning in shaping students’ cognitive outcomes in AI-assisted learning environments. The study contributes to a more nuanced understanding of how GenAI influences higher-order thinking and provides implications for promoting effective and responsible AI use in education.

## 1. Introduction

Amid the rapid development of science and technology, higher-order thinking skills have increasingly been recognized as essential competencies for students’ future employability and long-term professional competitiveness (Hague, 2024). Higher-order thinking skills are generally regarded as advanced cognitive competencies that involve critical thinking, creative thinking, and computational thinking, enabling individuals to effectively address complex problems and adapt to both academic and authentic real-world contexts (Rui et al., 2025). In educational settings, the cultivation of higher-order thinking skills is considered essential for enabling learners to move beyond rote memorization and procedural learning toward deeper conceptual understanding, reflective inquiry, and the transfer of knowledge across contexts (Loyens et al., 2023). Accordingly, identifying the key determinants and effective pedagogical approaches that facilitate the development of higher-order thinking skills has become a major concern among educational policymakers, researchers, and academic institutions worldwide.

In recent years, the widespread adoption of generative artificial intelligence (GenAI) in educational contexts has substantially reshaped students’ learning activities and the development of higher-order thinking skills (Kasneci et al., 2023; Lai et al., 2026). On the one hand, GenAI systems can provide learners with immediate, personalized, and extensive informational support. Such processes may encourage learners to consider problems from multiple perspectives and engage in more elaborative cognitive processing, thereby facilitating the development of higher-order thinking skills (Suriano et al., 2025). On the other hand, emerging evidence suggests that excessive reliance on GenAI may reduce learners’ engagement in deep learning and limit their use of higher-order cognitive processes (Tian & Zhang, 2025). These findings indicate that the effects of GenAI on higher-order thinking may be both facilitative and inhibitive, depending largely on how learners interact with AI-assisted systems. Therefore, merely examining the frequency of GenAI use may be insufficient for understanding its influence on students’ higher-order thinking development in educational settings.

From the perspective of self-regulated learning theory, learners actively regulate their cognitive, motivational, and behavioral processes throughout learning activities (Pintrich, 2004; Zimmerman, 2002). Within GenAI-assisted learning environments, such regulatory capacities may determine whether AI use promotes meaningful cognitive engagement or instead leads to passive cognitive offloading. Accordingly, self-regulated learning may function as a critical moderator in the relationship between GenAI use and the development of higher-order thinking skills. The present study therefore extends beyond merely measuring the frequency of students’ GenAI use by further examining their self-regulated learning during GenAI-assisted academic activities. Specifically, this study investigates how GenAI usage, together with self-regulated learning, relates to three core dimensions of higher-order thinking skills: critical thinking, creative thinking, and computational thinking.

### 1.1. The Relationship Between GenAI Usage and Higher-Order Thinking Skills

Higher-order thinking skills encompass multiple advanced forms of cognition, among which critical thinking, creative thinking, and computational thinking are widely recognized as core dimensions in contemporary educational research (Chen & Chi, 2022; Lewis & Smith, 1993). Critical thinking is generally conceptualized as a reflective and evaluative form of cognition involving the analysis, interpretation, and assessment of information, arguments, and evidence to support reasoned judgment and decision-making (Hwang et al., 2023). It requires individuals to engage in logical reasoning, evaluate the credibility and validity of information sources, identify assumptions and inconsistencies, and critically examine alternative perspectives before drawing conclusions. Creative thinking, by contrast, primarily reflects divergent and generative cognitive processes associated with the production of original, flexible, and contextually appropriate ideas (Bray et al., 2020). It involves the capacity to transcend conventional patterns of thought, establish novel conceptual associations, and generate innovative solutions to complex or ill-defined problems. Computational thinking represents a distinct form of higher-order cognition grounded in systematic and procedural approaches to problem-solving (Astor et al., 2026). It refers to the effective application of computational concepts and problem-solving strategies to address complex problems and facilitate knowledge transfer across diverse contexts (Tsarava et al., 2022). Unlike critical thinking, which centers on evaluative judgment, or creative thinking, which emphasizes novelty generation, computational thinking prioritizes logical organization and procedural reasoning in addressing complex problems.

The increasing integration of GenAI tools, such as ChatGPT-5, into higher education has generated substantial scholarly interest in their potential implications for students’ higher-order thinking skills. From the perspectives of cognitive load theory (Sweller, 1994) and cognitive offloading theory (Risko & Gilbert, 2016), the influence of GenAI on higher-order thinking may involve both facilitative and detrimental pathways. Cognitive load theory suggests that GenAI tools such as ChatGPT may reduce learners’ cognitive burden by assisting with information retrieval, organization, and task management (Suriano et al., 2025). By offloading lower-level or routine cognitive processes, learners may preserve working memory resources for more complex forms of cognition, including analysis, evaluation, reflection, and idea generation. In this sense, GenAI may function as a cognitive support tool that facilitates the development of higher-order thinking skills.

However, these theoretical perspectives also suggest potential risks associated with excessive reliance on GenAI responses. When learners habitually externalize cognitive processes to AI systems, they may reduce active engagement in deeper cognitive activities that are essential for higher-order thinking development (Tian & Zhang, 2025). Rather than supporting cognition, GenAI may replace independent reasoning, reflective judgment, and complex problem-solving, thereby weakening critical thinking, creative thinking, and computational thinking over time. Cognitive offloading theory further suggests that persistent dependence on external cognitive tools may foster passive learning tendencies and diminish learners’ willingness to engage in effortful cognitive processing (Chaudhry et al., 2023; Storm et al., 2017). Consequently, the effects of GenAI usage on higher-order thinking may not be uniformly positive or negative, but may instead depend on how learners regulate and cognitively engage with AI-assisted learning activities.

Recent empirical studies have suggested that the influence of GenAI usage on creative thinking and computational thinking may involve both facilitative effects and potential cognitive risks. On the one hand, empirical findings have shown that GenAI-assisted learning activities may enhance learners’ critical thinking by supporting evidence evaluation, reflective inquiry, and multi-perspective analysis (Fabio et al., 2025; Suriano et al., 2025). Existing studies have also reported that GenAI can facilitate creative thinking by promoting idea generation, cognitive flexibility, and the exploration of diverse solutions (Lee & Chung, 2024; Z. Zhou et al., 2025). In addition, research conducted in STEM and computing education has indicated that GenAI may support the development of computational thinking skills, including problem-solving efficiency, code comprehension, and algorithmic reasoning (Hsu & Hsu, 2025; Xu et al., 2026).

On the other hand, an increasing body of empirical evidence has raised concerns regarding the potential negative consequences of excessive reliance on GenAI. Studies on critical thinking have suggested that frequent dependence on AI-generated responses may reduce learners’ engagement in independent reasoning and critical evaluation of information (Tian & Zhang, 2025; Yurt & Kuşci, 2026). Similarly, empirical research on creative thinking has reported that AI-assisted tasks may decrease originality, weaken creative confidence, and increase the similarity of generated ideas (Habib et al., 2024; Moon et al., 2025). Moreover, scholars have argued that the use of GenAI in educational settings may foster “metacognitive laziness,” whereby learners become less inclined to actively monitor, evaluate, and regulate their own thinking processes (Fan et al., 2025; Ouaazki et al., 2026). Such reduced cognitive and metacognitive engagement may ultimately undermine the development of higher-order thinking skills. These findings imply that the cognitive effects of GenAI use may be shaped by the ways in which learners engage with and utilize AI-assisted learning tools.

Accordingly, the influence of GenAI usage on students’ higher-order thinking should not be viewed as inherently beneficial or detrimental. When learners engage with GenAI in a reflective and self-regulated manner, GenAI tools may support deeper cognitive engagement and intellectual exploration. However, excessive reliance on AI-generated responses or passive patterns of AI use may reduce independent cognitive effort and undermine the development of higher-order thinking skills (Strzelecki, 2024). In the present study, GenAI usage refers specifically to students’ academic engagement with emerging text-based generative AI tools (e.g., ChatGPT and similar large language model applications) during the current stage of GenAI adoption in higher education. This includes the use of GenAI for learning-related activities, such as completing assignments, supporting academic projects, generating ideas, and solving academic problems. Accordingly, the present study focuses on students’ current patterns of interaction with GenAI as an educational resource rather than general exposure to artificial intelligence technologies.

### 1.2. The Moderating Role of Self-Regulated Learning

Self-regulated learning refers to the process through which learners proactively regulate their cognition, motivation, behavior, and learning environment in pursuit of academic goals (Zimmerman, 2000, 2002). Rather than passively receiving information, self-regulated learners actively plan, monitor, and evaluate their learning activities and adjust their strategies in response to task demands and performance feedback. Self-regulated learning emphasizes learners’ active agency in the learning process and highlights the importance of self-monitoring, strategic action, and self-reflection in achieving effective learning outcomes (Panadero, 2017).

A growing body of research has demonstrated that self-regulated learning plays an important role in the development of higher-order thinking skills. The cognitive and metacognitive processes involved in self-regulation encourage learners to engage in deeper information processing, reflective inquiry, and strategic problem solving, all of which are fundamental components of higher-order thinking (Jia et al., 2019; Lebuda & Benedek, 2025). Studies have shown that learners with stronger self-regulatory capacities tend to exhibit higher levels of critical thinking, as effective monitoring and metacognitive regulation facilitate the evaluation and interpretation of information (X. Zhou et al., 2024). Similarly, self-regulated learning has been linked to creative thinking, with self-monitoring, strategy adaptation, and flexible learning approaches supporting the generation of novel and useful ideas (Callan et al., 2021). Research has also highlighted the positive contribution of self-regulated learning to computational thinking (Kong & Wang, 2024; Q. Li et al., 2025). Learners who effectively regulate their learning are more likely to exhibit stronger computational thinking abilities and employ more strategic approaches to problem solving. Consequently, self-regulated learning has been identified as an important factor supporting the development of critical thinking, creative thinking, and other advanced cognitive abilities.

Beyond its direct contribution to higher-order thinking, self-regulated learning may also influence how learners utilize and benefit from external learning resources and technological support. From the perspective of self-regulated learning theory, learning outcomes are shaped not only by the characteristics of learning environments and tools but also by learners’ ability to actively regulate their cognition, motivation, and behavior during the learning process (Panadero, 2017). In GenAI-assisted learning environments, students differ considerably in their capacity to critically evaluate AI-generated information, monitor their understanding, and strategically integrate AI support into their learning activities. Consequently, the influence of GenAI on higher-order thinking skills may vary as a function of learners’ self-regulatory capacities. Students with stronger self-regulatory capacities are more likely to use GenAI as a cognitive support tool that promotes deeper learning and intellectual engagement, whereas those with lower levels of self-regulation may be more susceptible to passive reliance on AI-generated outputs. Therefore, self-regulated learning may influence the extent to which GenAI use facilitates or constrains the development of higher-order thinking skills.

### 1.3. The Present Study

Although prior research has extensively examined the relationship between GenAI and higher-order thinking, most studies have focused primarily on the frequency of use or the extent of AI dependence (e.g., Abbas et al., 2024; Tian & Zhang, 2025). As a result, findings remain mixed, with some studies reporting cognitive benefits (Lee & Chung, 2024) and others suggesting potential negative consequences for higher-order thinking (Fan et al., 2025). These inconsistent findings imply that the effects of GenAI may depend on how learners regulate their use of AI-assisted learning tools. However, empirical evidence regarding the moderating role of self-regulated learning remains limited. To address this gap, the present study focuses on three core dimensions of higher-order thinking, i.e., critical thinking, creative thinking, and computational thinking, and examines whether self-regulated learning moderates the relationship between GenAI usage and these outcomes. Based on previous theoretical and empirical evidence, the present study proposes three hypotheses. First, given the potential facilitating and inhibiting effects of GenAI use on cognitive processing, we hypothesized that GenAI use would be significantly associated with students’ critical thinking, creative thinking, and computational thinking (H1). Second, based on self-regulated learning theory, we hypothesized that self-regulated learning would be positively associated with all three higher-order thinking skills (H2). Third, considering that students with different levels of self-regulated learning may engage with GenAI in different ways, we hypothesized that self-regulated learning would moderate the relationships between GenAI use and higher-order thinking skills (H3).

This study advances current understanding of the conditions under which GenAI facilitates or constrains higher-order thinking development and provides empirical evidence regarding the role of learner characteristics in shaping the cognitive consequences of GenAI use. Practically, the findings may inform the design of educational interventions and AI-supported learning environments that foster strategic, reflective, and self-regulated use of GenAI, thereby helping educators and policymakers maximize its cognitive benefits while reducing the risks associated with passive reliance on AI-generated outputs.

## 2. Methods

### 2.1. Participants

A total of 1067 undergraduate students from five universities were recruited for this study. Data from 43 participants (14 males and 29 females, Mage = 18.60 ± 0.79) who did not complete the higher-order thinking assessments (i.e., critical thinking, creative thinking, and computational thinking tasks) were excluded from the analyses. Among these participants, 17 were not invited to participate in the second-stage cognitive assessments due to invariant responding detected in the first stage, and 26 voluntarily withdrew from the study before completing the second-stage assessments. The final sample comprised 1024 participants (403 males and 621 females), with ages ranging from 17 to 25 years (*M* = 19.74, *SD* = 1.91). The study protocol was reviewed and approved by the Institutional Review Board of the corresponding author’s institution. All participants provided written informed consent prior to participation, after being informed of the study’s purpose, procedures, and their right to withdraw at any time without penalty. Participants received monetary compensation of 20 Chinese yuan for their participation.

### 2.2. Measure of GenAI Usage

The usage of GenAI among university students was assessed using the GenAI Usage Scale developed by Abbas et al. (2024). This instrument comprises eight items designed to capture the frequency and extent of GenAI use in academic contexts. An example item is: “I use GenAI for my course projects.” Participants responded using a six-point Likert scale, with higher scores indicating more frequent use of GenAI. The Chinese version of the scale was developed using a forward and backward translation approach. Initially, two bilingual researchers independently translated the original items into Chinese. Subsequently, an independent bilingual researcher who was unfamiliar with the original scale conducted a back-translation. Any inconsistencies between the original and translated versions were reviewed and resolved through discussion among the research team. A one-factor CFA model was conducted to examine the construct validity of the GenAI usage scale. The results indicated an acceptable model fit, χ^2^/df = 6.59, CFI = 0.97, TLI = 0.96, RMSEA = 0.074, SRMR = 0.034. In the present study, the scale demonstrated good internal consistency, with a Cronbach’s alpha of 0.88, suggesting that the instrument provides reliable measurement of GenAI usage among university students.

### 2.3. Measure of Self-Regulated Learning

Self-regulated learning in the context of GenAI use was assessed using the Self-Regulated Learning Scale developed by Ji et al. (2025). This instrument is designed to measure students’ ability to plan, monitor, and regulate their learning processes when engaging with GenAI tools. An example item is: “When using GenAI for learning, I set completion standards for my learning tasks.” The scale consists of 21 items spanning six dimensions: goal setting, task strategies, time management, self-evaluation, environment structuring, and help seeking. Each item is rated on a five-point Likert scale, with higher scores indicating greater self-regulated learning ability. Ji et al. (2025) demonstrated that the scale exhibits satisfactory reliability and validity in samples of university students. In the present study, the scale demonstrated excellent internal consistency, with a Cronbach’s alpha coefficient of 0.93, indicating that it provides a highly reliable measure of self-regulated learning in the context of GenAI usage. The Cronbach’s alpha coefficients for the six subscales were 0.82 for goal setting, 0.79 for task strategies, 0.81 for time management, 0.85 for self-evaluation, 0.78 for environment structuring, and 0.80 for help seeking, respectively. A CFA was conducted to examine the six-factor structure of the scale in the present sample. The hypothesized six-factor model demonstrated an acceptable-to-good fit to the data, χ^2^/df = 4.33, CFI = 0.95, TLI = 0.93, RMSEA = 0.057, and SRMR = 0.038, providing support for the proposed six-dimensional structure of this scale.

### 2.4. Measures of Higher-Order Thinking Skills

Critical thinking. Critical thinking skills were assessed using the Test of Critical Thinking Skills for Adults (TCTS-A; Yeh et al., 2001). The TCTS-A has been shown to possess satisfactory psychometric properties, with evidence of stable reliability and validity (Yeh et al., 2001). The instrument comprises 30 items designed to measure five core dimensions of critical thinking: identifying assumptions, inductive reasoning, deductive reasoning, argument analysis, and argument evaluation, with six items allocated to each dimension. Each item presents four response options, and responses are scored dichotomously based on accuracy. The test was administered with a time limit of 25 min. A total score was computed by summing the number of correct responses across all items, with higher scores indicating greater critical thinking ability. In the present study, the internal consistency of the TCTS-A total score was 0.70 in the sample of university students. The Cronbach’s alpha coefficients for the five subscales were 0.69 for identifying assumptions, 0.73 for inductive reasoning, 0.71 for deductive reasoning, 0.75 for argument analysis, and 0.66 for argument evaluation, respectively, indicating acceptable internal consistency across the subscales.

Creative thinking. The Alternate Uses Task (AUT) was employed to assess creative thinking. This task is widely recognized as a standard measure of divergent thinking (Boers et al., 2025; Urban et al., 2024). In the present study, participants were instructed to generate as many novel and unconventional uses as possible for each object within a five-minute time limit. A total of four items were used: brick, umbrella, newspaper, socks. Creative performance was evaluated across three dimensions: fluency, flexibility, and originality. All responses were independently scored by three trained raters. The scoring criteria were defined as follows: (1) Fluency was operationalized as the total number of acceptable responses produced by each participant. (2) Flexibility referred to the number of distinct conceptual categories represented in the responses. (3) Originality was assessed on a 5-point scale ranging from 1 (not original) to 5 (highly original), based on the criteria of uncommonness, remoteness, and cleverness (Liang et al., 2024). Inter-rater reliability was assessed using intraclass correlation coefficients (ICCs). A two-way random-effects model with absolute agreement and average measures was applied, as the creativity scores were calculated by averaging the ratings from three independent raters. The ICC values among the three raters were 1.00 for fluency, 0.94 for flexibility, and 0.81 for originality, indicating satisfactory reliability across all dimensions. The three AUT indicators, fluency, flexibility, and originality, were positively correlated with one another (*r* = 0.51–0.64, *p* < .001), indicating moderate-to-strong associations among the indicators and supporting their aggregation into a composite creative-thinking score. These indicators were standardized using z-scores and averaged to derive a composite creativity score. This composite score was subsequently used as the creativity outcome variable in the moderation analyses.

Computational thinking. Computational thinking skills were assessed using the Computational Thinking Task developed by P. Li (2024), which was specifically designed and validated for Chinese university students. The computational thinking skills were assessed using the Computational Thinking Task developed by P. Li (2024), which was specifically designed and validated for Chinese university students. The task was developed based on the computational thinking frameworks proposed by Brennan and Resnick (2012) and Cheng et al. (2023). It consists of 22 items, including multiple-choice and fill-in-the-blank questions, designed to assess three core dimensions of computational thinking: computational concepts, algorithmic thinking, and algorithmic problem-solving. Computational concepts refer to learners’ understanding of fundamental computational principles and knowledge required for developing computational solutions, such as iteration and parallelism. Algorithmic thinking refers to learners’ ability to construct computational algorithms for solving specific problems by organizing logical procedures and sequential steps. Algorithmic problem-solving emphasizes learners’ ability to explore and understand practical problems in depth and apply computational approaches to analyze, model, and solve these problems effectively. Representative sample items and their corresponding assessed dimensions are presented in Table 1. In the validation study, P. Li (2024) employed a Multidimensional Item Response Theory (MIRT) framework to examine the internal structure and item quality of the task. The model fit indices (AIC = 9148.172, BIC = 9738.938, CFI = 0.937, TLI = 0.917, RMSEA = 0.046) supported the hypothesized multidimensional structure of the assessment. Item difficulty and discrimination were evaluated based on MIRT parameters. The results showed that item difficulty values ranged from −2.42 to 2.65, indicating an acceptable level of difficulty. Moreover, all items demonstrated satisfactory discrimination, with MDISC values exceeding 0.5, suggesting that the items were effective in differentiating students’ computational thinking abilities. The above findings indicate that the measure is appropriate for use among Chinese university students. Each item has a single correct answer and is scored dichotomously, with one point awarded for each correct response. A composite score was calculated by summing all correctly answered items, with higher scores indicating greater computational thinking proficiency. In the present study, the scale demonstrated acceptable internal consistency, with a Cronbach’s alpha coefficient of 0.79.

### 2.5. Procedure and Data Analyses

GenAI usage and self-regulated learning were assessed using self-report scales, whereas critical thinking, creative thinking, and computational thinking were measured through performance-based assessments. To minimize potential fatigue effects, data collection was conducted across two separate sessions. In the first session, participants completed the measures of GenAI usage and self-regulated learning. In the second session, participants completed the three higher-order thinking assessments. To control for potential order effects, the tasks within each testing session were presented in a randomized order across participants. All assessments were completed within a one-week period.

Data analyses were conducted using IBM SPSS Statistics 22.0 and the PROCESS v4.1 macro. First, descriptive statistics, including means, standard deviations, skewness, and kurtosis, were computed to assess the distributional characteristics of the variables. Cronbach’s alpha coefficients were calculated to evaluate the internal consistency reliability of all scales. Second, Pearson correlation analyses were performed to assess the associations among GenAI usage, self-regulated learning, and higher-order thinking skills (i.e., critical thinking, creative thinking, and computational thinking). Third, multiple regression analyses were conducted to investigate the effects of GenAI usage and self-regulated learning on higher-order thinking skills. Age, gender, university, major, and discipline were included as covariates to account for potential demographic and educational-context differences that could confound the associations among GenAI use, self-regulated learning, and higher-order thinking skills. To test the moderating role of self-regulated learning, interaction terms were created after mean-centering the predictor variables to reduce potential multicollinearity. Finally, moderation analyses were conducted using PROCESS Macro Model 1. Significant interaction effects were further interpreted through simple slope analyses at low (−1 SD) and high (+1 SD) levels of self-regulated learning.

## 3. Results

### 3.1. Descriptive Statistics

The means, standard deviations, skewness, and kurtosis of GenAI usage, self-regulated learning, critical thinking, creative thinking, and computational thinking were presented in Table 2. All correlations among the variables were statistically significant (*p* < 0.05).

### 3.2. Hierarchical Regression Models

We constructed three separate linear regression models to examine whether self-regulated learning moderates the relationship between GenAI usage and three distinct higher-order cognitive skills: critical thinking, creative thinking, and computational thinking (Table 3). Residual diagnostics indicated no apparent violations of linearity or homoscedasticity based on visual inspection of standardized residual plots. Furthermore, all Cook’s distances were below 1.0, indicating no influential observations, and all variance inflation factors (VIFs) were below 2.0, indicating that multicollinearity was not a concern. The results showed that GenAI usage significantly predicted all three higher-order thinking skills. Specifically, GenAI usage was positively associated with critical thinking and computational thinking, but negatively associated with creative thinking. In addition, self-regulated learning emerged as a significant positive predictor of critical thinking, creative thinking, and computational thinking across all models. Furthermore, the interaction term (GenAI usage × self-regulated learning) was significant, indicating that the direction and strength of the association between GenAI usage and each cognitive skill depended on students’ level of self-regulated learning. Because three moderation models were tested across the three higher-order thinking outcomes, the Benjamini–Hochberg false discovery rate procedure was applied to the interaction terms. All moderation effects remained statistically significant after FDR correction. We further test the robustness of the moderation effect by adopting heteroskedasticity-robust (HC3) standard errors (see Supplementary Tables S1–S3). The GenAI × SRL interaction remains statistically significant for all three higher-order thinking outcomes, which supports the reliability of our moderation results.

Additional analyses were conducted to examine potential nonlinear associations between GenAI use and higher-order thinking outcomes. The quadratic effect of GenAI use and its interaction with SRL were nonsignificant across all three moderation models, suggesting no evidence of curvilinear relationships or nonlinear moderation effects.

**Figure 1:**
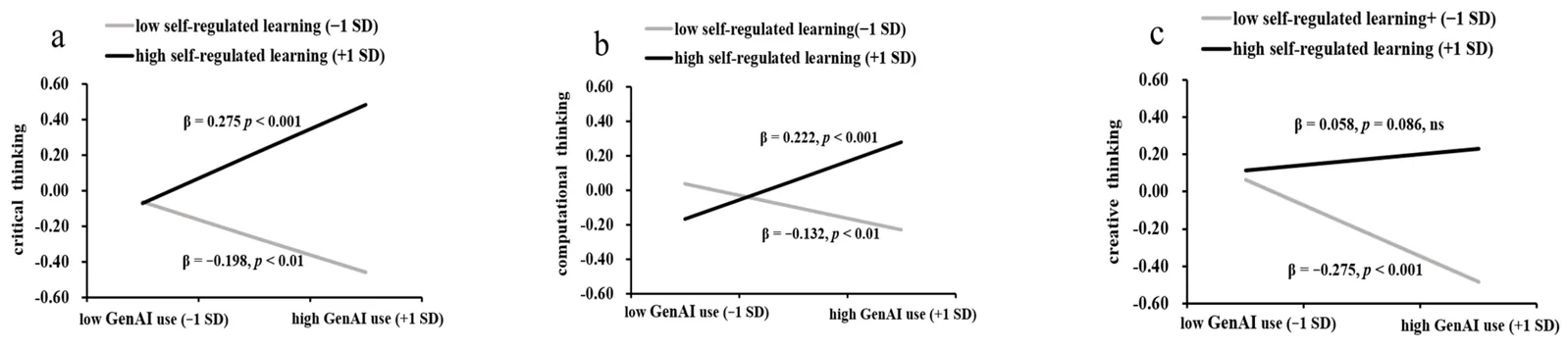
Simple slopes of GenAI usage predicting higher-order thinking skills at varying levels of self-regulated learning: (**a**) critical thinking; (**b**) computational thinking; (**c**) creative thinking.

Simple slope analyses were conducted to further explore the significant interaction effects. For critical thinking, as shown in Figure 1a, the results indicated that GenAI usage was negatively associated with critical thinking among students with low levels of self-regulated learning (−1 SD), β = −0.198, *t* = −5.227, *p* < .001, 95% CI [−0.272, −0.123], suggesting that reliance on GenAI may hinder critical thinking when self-regulatory skills are limited. In contrast, among students with high levels of self-regulated learning (+1 SD), GenAI usage positively predicted critical thinking, β = 0.275, *t* = 6.989, *p* < .001, 95% CI [0.198, 0.352], indicating that effective self-regulation enables students to leverage GenAI to enhance critical thinking. Johnson–Neyman analysis further indicated that the conditional effect of GenAI use became significantly negative when self-regulated learning was below −0.424 SD and significantly positive when self-regulated learning was above 0.087 SD.

As presented in Figure 1b, a similar moderation pattern was found for computational thinking. Specifically, GenAI usage was negatively associated with computational thinking at low levels of self-regulated learning, β = −0.132, *t* = −3.290, *p* < .01, 95% CI [−0.211, −0.053], whereas it positively predicted computational thinking among students with high self-regulated learning, β = 0.222, *t* = 5.312, *p* < .001, 95% CI [0.139, 0.304]. Johnson–Neyman analysis further indicated that the association between GenAI usage and computational thinking was significantly negative when self-regulated learning was below −0.646 SD and significantly positive when self-regulated learning exceeded 0.103 SD.

As shown in Figure 1c, the simple slope analyses for creative thinking illustrate the interaction between GenAI usage and self-regulated learning. At low levels of self-regulated learning, GenAI usage negatively predicted creative thinking, β = −0.275, *t* = −6.925, *p* < .001, 95% CI [−0.353, −0.197]. For students with high levels of self-regulated learning, the association between GenAI usage and creative thinking was not statistically significant, β = 0.058, *t* = 1.408, *p* = .159, 95% CI [−0.022, 0.139]. Johnson–Neyman analysis indicated that the negative association was significant when self-regulated learning was below 0.267 SD. Taken together, these results demonstrate that self-regulated learning not only influences the strength but also the direction of the relationship between GenAI usage and higher-order thinking skills, highlighting the critical role of self-regulatory processes in determining whether GenAI use supports or undermines students’ thinking ability.

## 4. Discussion

The present study examined the relationships between GenAI usage and three core higher-order thinking skills, i.e., critical thinking, creative thinking, and computational thinking, as well as the moderating role of self-regulated learning. Several important findings emerged. First, GenAI use was significantly associated with higher-order thinking skills, although the patterns of association varied across the three thinking skills. Second, self-regulated learning was positively associated with critical thinking, creative thinking, and computational thinking, highlighting its important role in fostering higher-order cognitive development. Third, self-regulated learning moderated the relationship between GenAI usage and higher-order thinking skills, indicating that the cognitive effects of GenAI may depend on learners’ ability to regulate their learning processes in AI-assisted learning environments.

A notable finding of the present study is that GenAI usage was positively associated with critical thinking and computational thinking, but negatively associated with creative thinking only among students with lower levels of self-regulated learning. This pattern provides empirical support for the proposition that GenAI may function as a double-edged tool in educational settings, offering cognitive benefits while simultaneously posing potential risks to certain aspects of higher-order thinking (Suriano et al., 2025). The positive associations with critical thinking and computational thinking are consistent with previous studies indicating that GenAI can facilitate analytical reasoning, problem solving, and complex cognitive processing (Fabio et al., 2025; Xu et al., 2026). In contrast, the negative association with creative thinking aligns with emerging evidence, suggesting that reliance on AI-generated content may reduce opportunities for original idea generation and independent creative exploration (Habib et al., 2024; Moon et al., 2025). Taken together, these findings are consistent with prior research reporting mixed effects of GenAI use and suggest that its influence on higher-order thinking cannot be characterized as uniformly beneficial or detrimental.

Another important finding is that self-regulated learning was positively associated with critical thinking, creative thinking, and computational thinking. This finding is consistent with self-regulated learning theory, which emphasizes learners’ active involvement in planning, monitoring, and evaluating their cognitive and behavioral activities during learning (Zimmerman, 2000, 2002). Through these processes, individuals may be more likely to engage in sustained cognitive effort, reflective inquiry, and strategic adaptation, which are associated with stronger higher-order thinking skills. The present findings also accord with previous empirical evidence highlighting the contribution of self-regulation learning to advanced cognitive development (Callan et al., 2021; X. Zhou et al., 2024). Learners who effectively regulate their learning tend to engage more actively in evaluating information, generating and refining ideas, and adopting strategic approaches to complex problem solving (Wang et al., 2024). These regulatory processes have been linked to stronger critical thinking, enhanced creative performance, and higher levels of computational thinking in prior research (Callan et al., 2021; Kong & Wang, 2024; X. Zhou et al., 2024). Collectively, the findings suggest that self-regulated learning serves as an important cognitive resource associated with diverse higher-order thinking skills.

More importantly, the present study found that self-regulated learning significantly moderated the relationships between GenAI usage and all three higher-order thinking skills. Although empirical research directly examining this moderating mechanism remains scarce, the finding is broadly consistent with previous studies emphasizing the importance of self-regulation in AI-assisted learning environments (Mohebbi, 2025; Z. Zhou et al., 2025). Prior research has shown that learners with stronger self-regulatory capacities are more likely to engage strategically with AI-supported learning resources and derive greater educational benefits from technology-enhanced learning environments (Wu & Chiu, 2025; Yang, 2026). The present study extends this line of research by demonstrating that self-regulated learning is not only associated with learning outcomes directly but also moderates the associations between GenAI use and higher-order thinking outcomes. The current findings extend this line of research by demonstrating that self-regulated learning may also shape the cognitive outcomes associated with GenAI use. One possible explanation is that self-regulated learners tend to treat GenAI as a cognitive support tool rather than a substitute for thinking. Through ongoing monitoring and evaluation, they are more likely to critically assess AI-generated information, compare alternative perspectives, and integrate external support into their own reasoning processes. In contrast, learners with lower levels of self-regulated learning may rely more passively on AI-generated responses, thereby reducing opportunities for independent cognitive engagement and deeper knowledge construction. Therefore, self-regulated learning may function as an important boundary condition that helps explain when GenAI use is associated with more favorable or less favorable higher-order thinking outcomes.

Several limitations should be acknowledged when interpreting the findings of this study. First, creative thinking was assessed only using the AUT. Although the AUT is one of the most widely used measures of creative thinking and captures a core component of creative thinking (i.e., divergent thinking), it does not fully represent all aspects of creative thinking. Future research should incorporate multiple creativity measures to further examine the relationship between GenAI usage and diverse facets of creative thinking. Second, the cross-sectional design of the present study limits the ability to determine the temporal dynamics and causal directions among GenAI usage, self-regulated learning, and higher-order thinking skills. Although the findings provide evidence for the moderating role of self-regulated learning, it remains unclear whether these relationships remain stable or change as students’ experiences with GenAI develop over time. Future research should employ longitudinal or experimental designs to examine the developmental trajectories and causal mechanisms underlying the relationships between GenAI usage and higher-order thinking skills. Third, the present study was conducted among Chinese university students, which may limit the generalizability of the findings. Moreover, participants were recruited from five universities and represented four disciplinary areas (natural sciences, engineering and technology, medicine, and social sciences). Although the inclusion of students from multiple institutions and disciplines provides some diversity in educational contexts, the findings may not fully generalize to students from other types of institutions, academic disciplines, or educational systems. Future research should examine whether the observed relationships among GenAI use, self-regulated learning, and higher-order thinking can be replicated across diverse populations, institutional contexts, disciplinary domains, cultural backgrounds, and educational systems. Fourth, the current measure of GenAI use has limitations in capturing the temporal and qualitative dimensions of students’ engagement with GenAI. Specifically, the measure primarily assesses usage frequency and does not specify an explicit retrospective timeframe (e.g., the past month or semester), which may introduce ambiguity in how participants interpret and report their frequency of use. Moreover, students with similar levels of GenAI use may adopt different strategies, ranging from passive reliance on AI-generated outputs to active use for idea exploration and reflection. Future studies should incorporate behavior-based measures, explicit time-anchored assessments, or qualitative methods to capture variations in GenAI usage patterns and better understand how self-regulated learning shapes these interactions. Finally, the present study focused on the moderating role of self-regulated learning but did not examine the underlying mechanisms. Future research could explore potential mediating processes, such as metacognitive monitoring, cognitive effort, and motivational processes, to provide a more comprehensive understanding of how self-regulated learning shapes the associations between GenAI use and higher-order thinking.

The present study contributes to the growing literature on GenAI and higher-order thinking by demonstrating that the cognitive consequences of GenAI use are not uniformly positive or negative. By simultaneously examining critical thinking, creative thinking, and computational thinking, the findings provide a more nuanced understanding of how GenAI relates to different forms of higher-order cognition. Furthermore, this study extends self-regulated learning theory to the context of GenAI-assisted learning by identifying self-regulated learning as an important boundary condition in the relationship between GenAI usage and higher-order thinking skills. The findings suggest that learners’ self-regulatory capacities may influence how effectively they leverage AI-generated support for cognitive development. From a practical perspective, the results highlight the importance of fostering self-regulated learning skills alongside the integration of GenAI in educational settings. Educational institutions and instructors should not only encourage the effective use of GenAI tools but also cultivate learners’ abilities to critically evaluate, monitor, and strategically utilize AI-generated information. In particular, educators should provide explicit guidance on responsible and reflective GenAI use, helping students distinguish between passive reliance on AI-generated outputs and active engagement with AI as a cognitive support tool. Future educational technologies may consider developing GenAI systems embedded with scaffolds that promote self-regulated learning, such as goal-setting prompts, AI-output evaluation mechanisms, reflective questioning, and adaptive feedback. Such systems could support students in monitoring their learning processes, critically evaluating AI-generated information, and strategically regulating their interactions with GenAI, thereby reducing passive reliance and promoting higher-order thinking development.

## 5. Conclusions

This study examined the relationships between GenAI use and three higher-order thinking skills—critical thinking, creative thinking, and computational thinking—as well as the moderating role of self-regulated learning. The findings revealed that GenAI use was positively associated with critical thinking and computational thinking but negatively associated with creative thinking, highlighting the complex and multifaceted influence of GenAI on students’ cognitive development. In addition, self-regulated learning was positively related to all three higher-order thinking skills and moderated the relationships between GenAI use and these cognitive outcomes. These findings suggest that the educational value of GenAI depends not only on its use but also on learners’ ability to regulate their learning processes. Promoting self-regulated learning may therefore be essential for maximizing the benefits of GenAI while reducing its potential risks for higher-order thinking development.

## Figures and Tables

**Table 1:** Mapping of Representative Sample Items to Computational Thinking Dimensions.

Representative Sample Item		Assessed Computational Thinking Dimensions		
		Computational Concepts	Algorithmic Thinking	Algorithmic Problem-Solving
1. Please identify the function of the algorithm represented by the flowchart below.		√	√	
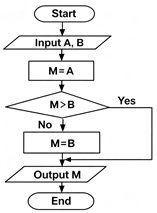	A. Sorting in descending order B. Finding the maximum value C. Swapping and outputting D. Making a judgment			
2. There are 25 questions in a mathematics competition. The scoring rules are as follows: 4 points are awarded for each correct answer, 0 points for each unanswered question, and 2 points are deducted for each incorrect answer. Xiao Ming received a total score of 90 points. How many questions did Xiao Ming answer correctly? A.22 B.23 C.24 D.25			√	√
3. Performing cuts on a rectangular chessboard: each cut is made in a direction parallel to one of the sides of the rectangle. What is the maximum number of pieces into which the board can be divided after n cuts? For example, when n = 3, the board can be divided into a maximum of 6 pieces. When n = 37, the board can be divided into a maximum of ______ pieces.			√	√

**Table 2:** Descriptive statistics and bivariate correlations of all variables.

Measure	Skewness	Kurtosis	*M*	*SD*	1	2	3	4
1.GenAI usage	0.13	−0.28	23.24	6.44	—			
2.Self-regulated learning	0.11	0.67	68.68	11.83	0.12 ***	—		
3. Critical thinking	0.13	−0.21	19.16	2.63	0.11 ***	0.24 ***	—	
4. Creative thinking	−0.41	0.93	36.23	4.90	−0.09 **	0.17 ***	0.14 ***	—
5. Computational thinking	0.09	−0.59	11.39	3.61	0.08 *	0.07 *	0.27 ***	0.12 ***

*Note.* * *p* < 0.05. ** *p* < 0.01.*** *p* < 0.001.

**Table 3:** Regression models on higher-order thinking skills.

Outcomes	Predictors	β	SE	*t*	VIF	
Critical thinking	Age	0.184	0.033	5.614 ***	1.282	*R*^2^ = 0.178
	Gender	0.164	0.032	5.133 ***	1.237	*F* = 18.215 ***
	GenAI usage	0.036	0.030	1.286	1.113	*ΔR*^2^ = 0.074
	Self-regulated learning	0.239	0.029	8.185 ***	1.021	
	GenAI use × Self-regulated learning	0.280	0.029	9.519 ***	1.008	
Creative thinking	Age	0.059	0.035	1.697	1.282	*R*^2^ = 0.089
	Gender	0.038	0.034	1.130	1.237	*F* = 8.187 ***
	GenAI usage	−0.107	0.032	−3.341 **	1.113	*ΔR*^2^ = 0.037
	Self-regulated learning	0.194	0.031	6.304 ***	1.021	
	GenAI use × Self-regulated learning	0.197	0.026	7.577 ***	1.008	
Computational thinking	Age	0.144	0.035	4.161 ***	1.282	*R*^2^ = 0.085
	Gender	0.065	0.034	1.940	1.237	*F* = 7.811 ***
	GenAI usage	0.037	0.032	1.137	1.113	*ΔR*^2^ = 0.041
	Self-regulated learning	0.087	0.031	2.828 **	1.021	
	GenAI use × Self-regulated learning	0.209	0.026	8.038 ***	1.008	

*Note.* Institution and discipline were dummy-coded and included as control variables in all models. None of the dummy-coded indicators showed significant effects and are not displayed in the table for parsimony. Standardized regression coefficients (β) are reported. ** *p* < 0.01.*** *p* < 0.001.

## Data Availability

The data presented in this study are available on request from the corresponding author due to privacy restrictions.

## Supplementary Materials

The following supporting information can be downloaded at: https://www.mdpi.com/article/10.3390/jintelligence14090208/s1, Table S1: Full regression results and HC3 robustness checks of the moderation effect for critical thinking; Table S2: Full regression results and HC3 robustness checks of the moderation effect for creative thinking; Table S3: Full regression results and HC3 robustness checks of the moderation effect for computational thinking.
