# Supplementary material for: Enhancing or Hindering? The Moderating Role of Self-Regulated Learning in the Relationship Between GenAI Usage and Higher-Order Thinking Skills Among Chinese University Students
Source: J Intell. 2026 Sep 1;14(9):208. doi: 10.3390/jintelligence14090208 (PMC13608059; doi:10.3390/jintelligence14090208)
Supplement: Supplementary file 1 [file jintelligence-14-00208-s001.zip › jintelligence-4398120-supplementary.pdf]

Supplementary Table S1

Full regression results and HC3 robustness checks of the moderation effect for critical thinking

Predictors	Model1 β	Model2 β	HC3 SE	HC3 *p*
Age	.175***	.184***	.034	<.001
Gender	.145***	.164***	.033	<.001
**University (ref. = University A)**				
University B	.128	.152	.097	.116
University C	.117	.118	.082	.148
University D	.081	.090	.074	.224
University E	.058	.071	.041	.084
**Discipline (ref. = Social Sciences)**				
Natural Sciences	.121	.167	.116	.149
Engineering & Technology	.067	.086	.082	.296
Medicine	.077	.084	.087	.334
GenAI usage	.029	.036	.036	.311
Self-regulated learning	.221***	.239***	.031	<.001
GenAI use × Self-regulated learning		.280***	.035	<.001
R^2	.104	.178		
ΔR^2		.074		

Note. Standardized regression coefficients (β) are reported. Model 1 includes age, gender, university indicators, discipline indicators, GenAI usage, and self-regulated learning. Model 2 additionally includes the GenAI use × Self-regulated learning interaction term. ΔR^2 represents the incremental variance explained by adding the GenAI use × Self-regulated learning interaction term to Model 1. HC3 SE and *p* values are based on heteroskedasticity-robust standard errors for Model 2. University A and Social Sciences were used as the reference categories. *$p < .05$. **$p < .01$.***$p < .001$.

Supplementary Table S2

Full regression results and HC3 robustness checks of the moderation effect for creative thinking

Predictors	Model1 β	Model2 β	HC3 SE	HC3 *p*
Age	.052	.059	.034	.086
Gender	.053	.038	.034	.257
**University (ref. = University A)**				
University B	.010	.003	.096	.717
University C	.011	.009	.081	.915
University D	.045	.052	.081	.523
University E	.001	.008	.046	.867
**Discipline (ref. = Social Sciences)**				
Natural Sciences	.039	.018	.114	.876
Engineering & Technology	.008	.021	.089	.811
Medicine	.025	.030	.044	.487
GenAI usage	-.112**	-.107**	.037	.003
Self-regulated learning	.181***	.194***	.035	<.001
GenAI use × Self-regulated learning		.197***	.034	<.001
R^2	.052	.089		
ΔR^2		.037		

Note. Standardized regression coefficients (β) are reported. Model 1 includes age, gender, university indicators, discipline indicators, GenAI usage, and self-regulated learning. Model 2 additionally includes the GenAI use × Self-regulated learning interaction term. ΔR^2 represents the incremental variance explained by adding the GenAI use × Self-regulated learning interaction term to Model 1. HC3 SE and *p* values are based on heteroskedasticity-robust standard errors for Model 2. University A and Social Sciences were used as the reference categories. *p < .05. **p < .01.***p < .001.

Supplementary Table S3

Full regression results and HC3 robustness checks of the moderation effect for computational thinking

Predictors	Model1 β	Model2 β	HC3 SE	HC3 p
Age	.137***	.144***	.036	<.001
Gender	.051	.065	.036	.067
**University (ref. = University A)**				
University B	.185	.158	.110	.149
University C	.166	.167	.098	.090
University D	.155	.162	.087	.062
University E	.138	.148	.091	.104
**Discipline (ref. = Social Sciences)**				
Natural Sciences	.107	.063	.133	.636
Engineering & Technology	.193	.197	.118	.096
Medicine	.183	.174	.142	.221
GenAI usage	.031	.037	.034	.280
Self-regulated learning	.074*	.087**	.035	.014
GenAI use × Self-regulated learning		.209***	.032	<.001
R^2	.044	.085		
ΔR^2		.041		

Note. Standardized regression coefficients (β) are reported. Model 1 includes age, gender, university indicators, discipline indicators, GenAI usage, and self-regulated learning. Model 2 additionally includes the GenAI use × Self-regulated learning interaction term. ΔR^2 represents the incremental variance explained by adding the GenAI use × Self-regulated learning interaction term to Model 1. HC3 SE and p values are based on heteroskedasticity-robust standard errors for Model 2. University A and Social Sciences were used as the reference categories. *p < .05. **p < .01.***p < .001.
